# Moyamoya Disease in Pregnancy: A Case Report and Review of the Literature

**DOI:** 10.7759/cureus.93567

**Published:** 2025-09-30

**Authors:** George Mpourazanis, Zoi Anastasiadi, Elisavet Melissi, Petros Papalexis, Michaela Papadatou, Athina Veneti, Apostolos Ntanasis, Magdalini Aliri, Michail Billis, Anastasia Zagaliki, Christos Akrivis, Panagiotis Tsirkas

**Affiliations:** 1 Department of Obstetrics and Gynecology, General Hospital of G.Hatzikosta Ioannina, Ioannina, GRC; 2 Department of Anesthesiology, General Hospital of G.Hatzikosta Ioannina, Ioannina, GRC; 3 Unit of Endocrinology, First Department of Internal Medicine, Laiko General Hospital, National and Kapodistrian University of Athens, Athens, GRC; 4 Department of Obstetrics and Gynecology, Laiko General Hospital of Athens, Athens, GRC; 5 Department of Surgery, General Hospital of G.Hatzikosta Ioannina, Ioannina, GRC

**Keywords:** mmd, moyamoya and cesarean section, moyamoya disease and syndrome, moyamoya disease (mmd), pregnancy and moyamoya

## Abstract

Moyamoya disease (MMD) is a rare cerebrovascular disorder characterized by the constriction or obstruction of the internal carotid and anterior cerebral arteries bilaterally, leading to either ischemic or hemorrhagic events.

A 28-year-old Caucasian female with a history of bilateral MMD was admitted for a scheduled cesarean delivery at 38 weeks of pregnancy. The management around the surgery involved stopping aspirin, closely monitoring hemodynamics, and administering general anesthesia. A healthy male baby was delivered via cesarean section, and both the mother and infant were discharged on the third day without any complications.

This case represents the first recorded instance of MMD during pregnancy in Greece. Achieving successful results is possible through early identification, tailored delivery planning, and collaborative care among obstetrics, neurology, and anesthesia teams. Additional multicenter research is necessary to create standard management protocols for this high-risk patient group.

## Introduction

Moyamoya disease (MMD) is a rare vascular condition affecting the brain, characterized by the progressive constriction or obstruction of the internal carotid and anterior cerebral arteries on both sides. This disorder can result in either ischemic or hemorrhagic symptoms, with children frequently exhibiting typical ischemic signs during their first episodes [1].

The incidence of MMD differs by region and race, with higher rates observed in Asia as compared to Europe, the Americas, Africa, and Latin America. Japan exhibits the highest prevalence, as indicated by a national survey conducted in 2003, which revealed a rate of 6.03 per 100,000 people and an annual incidence of 0.54 per 100,000 [2]. MMD is more prevalent in women, and the symptoms typically appear in their 20s or 30s, a time that may overlap with childbearing. As a result, it is fairly common for MMD to be identified during pregnancy or shortly after childbirth [3].

The exact cause of MMD is still not entirely understood. It is primarily regarded as a nonspecific immunoinflammatory condition that results in the persistent proliferation of the intima, the movement of smooth muscle cells, and the narrowing of the terminal intracranial section of the internal carotid artery, ultimately leading to blockage [4]. In children, the condition primarily appears as inadequate blood flow to the brain, resulting in temporary neurological dysfunction, sensory issues, seizures, headaches, and involuntary choreiform movements, with 80.5% of patients with Moyamoya experiencing motor function deficits. In adults, the issue mainly manifests as cerebral hemorrhage, which can include intracerebral, intraventricular, and subarachnoid hemorrhages. Symptoms may comprise headaches, loss of consciousness, hemiplegia, and sensory disturbances [5,6].

There are no standard protocols for handling pregnancy and childbirth in women diagnosed with MMD, and over 70% of pregnant individuals with MMD opt for cesarean delivery to minimize the likelihood of intracerebral hemorrhage or cerebral infarction [7]. Different surgical methods, such as superficial temporal artery (STA), encephaloduroarteriosynangiosis (EDAS), and emergency medical services (EMS), have been created to bypass the delicate MMV; however, their effectiveness in reducing morbidity and mortality in pregnant women has not been established through prospective studies [8]. In a cohort of women diagnosed with MMD caused by cerebrovascular incidents during pregnancy, 57.2% received neurosurgical intervention, and 80% had cesarean deliveries, resulting in maternal and fetal mortality rates of 13.6% and 23.5%, respectively [9].

We describe the case of a 29-year-old woman who is currently 38 weeks pregnant and underwent a scheduled cesarean delivery. Her medical history indicates that she was diagnosed with bilateral MMD when she was 13 years old. Since her diagnosis, she has had two craniotomies and bilateral cerebral revascularizations. This is a unique case of MMD occurring during pregnancy in Europe, making it noteworthy to describe.

## Case presentation

A 28-year-old Caucasian woman who is pregnant and at 38 weeks and 2 days of gestation arrived at the outpatient gynecological clinic for admission and to schedule a cesarean section due to MMD. Upon admission, the patient's vital signs were recorded as blood pressure at 140/80 mmHg, a temperature of 36.5 °C, a pulse rate of 72 beats per minute, a respiratory rate of 16 breaths per minute, and an oxygen saturation level of 98%. Her gynecological history indicates that menarche started at age 13, with menstrual cycles occurring every 28 to 30 days and lasting 4 to 5 days. A Pap smear conducted one year ago returned normal results.

**Figure 1:**
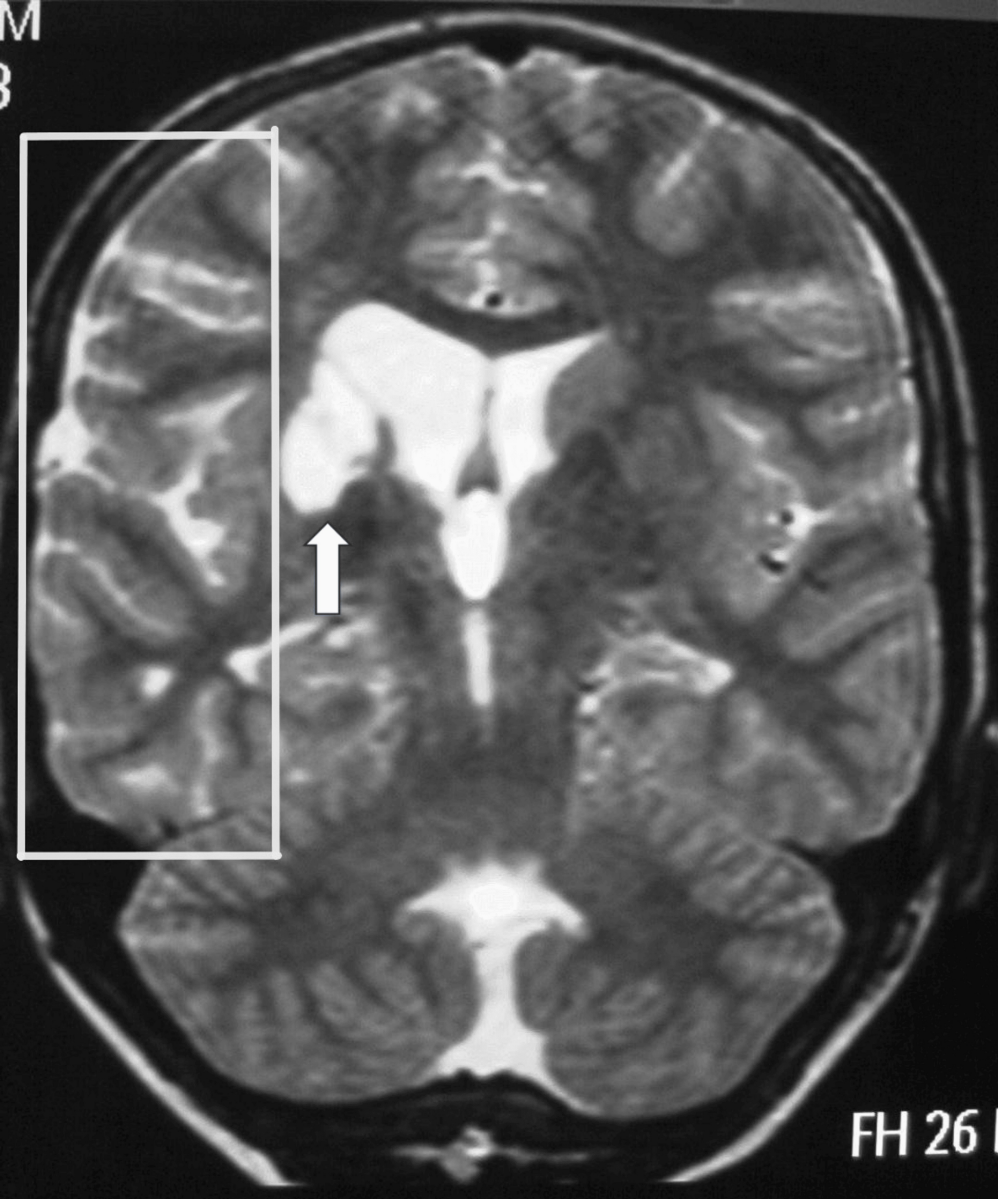
Brain MRI (axial T2-phase) of a female patient with bilateral Moyamoya disease, showing chronic ischemic infarcts and encephalomalacic changes predominantly in the right hemisphere (white outline), corresponding to the ischemic strokes she suffered at the age of 13. The white arrow highlights the periventricular ischemic lesion adjacent to the lateral ventricle. MRI: magnetic resonance imaging

**Figure 2:**
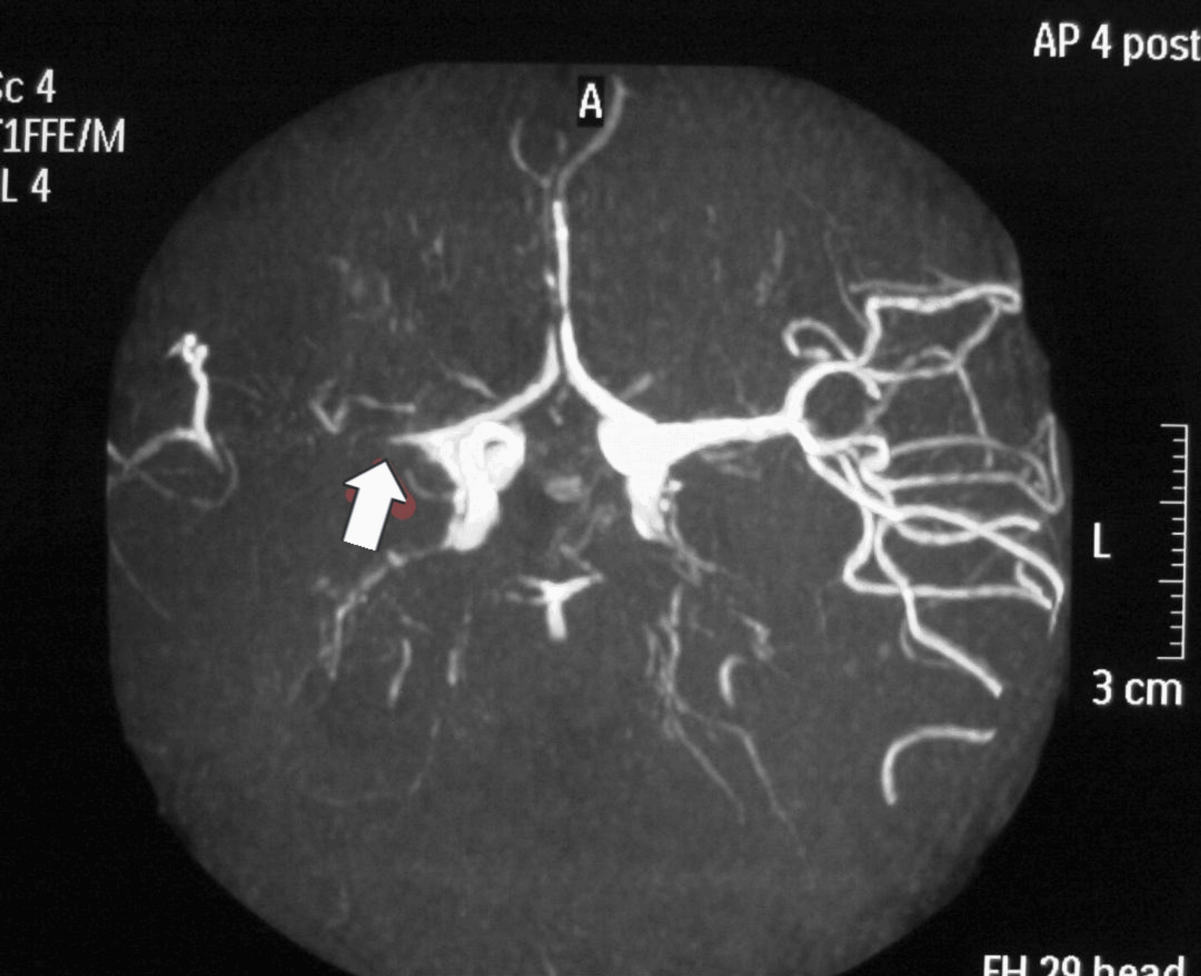
Magnetic resonance angiography (MRA) demonstrating marked narrowing and occlusion of the terminal portion of the right internal carotid artery (white arrow), with the formation of collateral “Moyamoya” vessels. This angiographic pattern is typical for Moyamoya disease and corresponds to the patient’s clinical history of progressive bilateral involvement, treated with staged craniotomy and cerebral angioplasty.

Based on her medical history, the patient has been diagnosed with hypothyroidism and is currently on a regimen of levothyroxine at a dose of 50 micrograms. She was first diagnosed with MMD bilateral at the age of 13, during which she experienced ischemic strokes and infarcts in the right side of her brain. The diagnosis was confirmed through digital angiography, leading to a craniotomy followed by right cerebral angioplasty. Her recovery was very successful, and her clinical condition improved significantly, with no impact on her speech or movement. At the age of 17, 4 years later, she had another procedure, this time on the left side, involving a craniotomy and left cerebral angioplasty. Since she was 13, she has been taking aspirin at a dose of 100 mg daily. The relevant characteristic imagings of the patient are described in Figures 1, 2.

During her pregnancy, there were no signs of her illness. Her medication regimen during this time included 100 mg of aspirin and 50 mg of levothyroxine. Aspirin was stopped five days prior to the scheduled cesarean section. The laboratory findings were noteworthy (Table 1).

**Table 1:** Preoperative and postoperative laboratory results WBC: white blood cell; LYMPH: lymphocyte; HGB: hemoglobin; HCT: hematocrit; INR: international normalized ratio; aPTT: activated partial thromboplastin time; PLT: platelet; CRP: C-reactive protein; AST: aspartate transferase; ALT: alanine transaminase; GGT: gamma-glutamyl transferase; ALP: alkaline phosphatase; ALB: albumin; GLC: glucose; TPR: total protein; UA: uric acid; URE: urea; CRE: creatinine; K+: potassium; Na+: sodium; TSH: Thyroid-stimulating hormone; FREE T4: free thyroxine; T3: triiodothyronine

Parameter	Day 0 (admission and operation)	Day 1	Day 3 (exit day)	40 days follow-up	Reference values
WBC	6.67 k/μL	15.49 k/μL	12.46 k/μL	6.50 k/μL	4-11 k/μL
Neutrophils	69%	94.1%	80.7%	55%	40-75%
LYMPH	20.1%	4.8%	13.5%	28%	20-45%
HBG	9.3 g/dl	10.6 g/dl	9.3 g/dl	12 g/dl	11.8-17.8 g/dl
HCT	30.0%	33.1%	30.1%	38%	36-52%
INR	0.95	0.98	0.94	-	0.8-1.2
aPTT	32.1 sec	22.40 sec	-	-	26-36 sec
PLT	354 k/μl	342 k/μl	358 k/μl	350 k/μl	140-450 k/μl
GLC	86 mg/dl	70 mg/dl	79 mg/dl	120 mg/dl	70-115 mg/dl
URE	40 mg/dl	15 mg/dl	19 mg/dl	44 mg/dl	10-50 mg/dl
CRE	1.06 mg/dl	0.89 mg/dl	0.73 mg/dl	0.94 mg/dl	0.5-1.1 mg/dl
K+	3.8 mmol/l	3.5 mmol/l	4.1 mmol/l	4 mmol/l	3.5-5.1 mmol/l
Na+	138 mmol/l	140 mmol/l	136 mmol/l	138 mmol/l	136-146 mmol/l
TPR	6.6 g/dl	6.3 g/dl	6.3 g/dl	7 g/dl	6.2-8.4 g/dl
ALB	3.1 g/dl	3.5 g/dl	3.0 g/dl	3.9 g/dl	3.5-5.1 g/dl
AST	18 U/L	22 U/L	20 U/L	17 U/L	5-33 U/L
ALT	20 IU/l	21 IU/l	23 IU/l	18 IU/l	5-32 IU/l
GGT	25 IU/l	15 IU/l	25 IU/l	24 IU/l	5-31 IU/L
UA	5.6 mg/dl	2.5 mg/dl	2 mg/dl	2.5 mg/dl	2.3-6.1 mg/dl
TSH	7.5 mlU/L	-	2.9 mlU/L	2.2 mlU/L	0.35-4.20 mlU/L
FREE T4	11 pmol/L	-	16 pmol/L	17.5 pmol/L	12-22 pmol/L
T3	1.15 nmol/L	-	1.7 nmol/L	1.8 nmol/L	1.3-3.1 nmol/L

In the operating room, the patient was positioned supine under general anesthesia. A male infant weighing 3280 grams was delivered. The uterus was sutured in two layers, and hemostasis was confirmed throughout the entire surgical area. The examination of the ovaries and fallopian tubes revealed that they were normal. The layers of the abdominal wall were carefully returned to their proper anatomical arrangement, and the skin was closed with a continuous intradermal stitch. During her time in the hospital, the patient experienced no neurological problems. On the third day postoperatively, both the patient and her infant were discharged from the gynecology ward. During the follow-up visit at the outpatient clinic for the gynecology department after 40 days, the patient was reported to be in overall good health. The three-month follow-up appointment with the neurologist showed stable results, with no indications of MMD returning.

## Discussion

MMD is a rare brain condition causing progressive obstruction of the internal carotid and anterior cerebral arteries, causing ischemic or hemorrhagic symptoms [1]. A case study involving MMD demonstrated that ultrasound-assisted spinal anesthesia is a safe, effective, and patient-focused approach to cesarean delivery in women with cerebrovascular conditions [10]. In a retrospective study of a case series, Dan Li and colleagues found that pregnant women with MMD who received 100 mg of aspirin daily and 20 mg of atorvastatin and who had left superficial temporal artery branch patching prior to a cesarean section under epidural anesthesia achieved favorable pregnancy outcomes that can be enhanced through effective multidisciplinary collaboration [11].

A 21-year study conducted at a single institution included 13 pregnant women diagnosed with MMD, with an average age of 30. The patients with MMD experienced transient ischemic attacks, infarctions, and headaches. Cerebral bypass was the chosen treatment for this condition. The findings of this study indicated that women with MMD experience a higher occurrence of hypertensive disorders compared to those with normal pregnancies and recommended that strict management of blood pressure should be implemented in MMD patients during pregnancy and the postpartum period [12]. A population-based study conducted across South Korea revealed that 412 women were diagnosed with MMD, with an average age of 35 years. The study found that having MMD was linked to a heightened risk of cerebrovascular disease (CVD) following delivery, with an adjusted hazard ratio of 37.42 and a 95% confidence interval (CI) of 17.50 to 80.02 within a period of 2.3 years, after controlling for factors such as pregnancy-induced hypertension, gestational diabetes mellitus, pregestational diabetes, and chronic hypertension [13].

A retrospective analysis of 20 pregnant women with MMD revealed that intracranial hemorrhage, especially around 24 weeks, frequently occurs during the antepartum phase, whereas cerebral infarction tends to be more prevalent after childbirth [14]. Jung et al. conducted a study over 8 years, which included 28 pregnant women diagnosed with MMD. The average age was 31.9, and all had previously undergone cephaloduroarterio-synangiosis and external ventricular drainage. Among these women, 25 opted for cesarean delivery, while only 2 experienced vaginal birth. The findings suggest that pregnant women with MMD should carefully manage blood pressure, avoid hyperventilation, and select the most appropriate delivery and anesthesia options to minimize the risk of negative postpartum outcomes [15].

Based on a national survey conducted in Japan, a questionnaire was distributed to 554 women diagnosed with MMD. The average age was 43.1, and these women presented initial signs of MMD, such as transient ischemic attacks and hemorrhages. They had received bypass surgery as treatment for their MMD. The results from this questionnaire indicated that a diagnosis of MMD lowers the risk of perinatal neurological incidents, but if the disease remains undiagnosed during pregnancy, severe complications, especially intracranial hemorrhage, can arise [16]. All relevant studies are mentioned in Table 2.

**Table 2:** Studies on Moyamoya in pregnancy

Authors/Publication Year/Citation	Type of Study	Number of Patients	Mean Age	Clinical Symptoms	Diagnostic Examination	Gestational Weeks	Therapy for Moyamoya	Type of Anesthesia	Delivery Method	Results
Jiang et al. 2025 [10]	Case report	1	28	Headache	Magnetic resonance angiography	37	Left superficial temporal artery-middle cerebral bypass	Spinal anesthesia	Cesarean section	Ultrasound-assisted SA is a safe, effective, and patient-centered method of cesarean birth in women who have cerebrovascular disease
Dan Li et al. 2024 [11]	Retrospective analysis of case series	3	27 to 41	Headache, Numbness in the right face and hand, Vomiting, Coma	Computed tomography scan, Magnetic resonance imaging, Digital Subtraction Angiography	39+, 38+, 32+,	Aspirin 100mg qd, Atorvastatin 20 mg qn, Probeco 0.5 g bid, Left superficial temporal artery branch patching	Epidural anesthesia	Cesarean section	Positive pregnancy outcomes can be maximized through efficient multidisciplinary teamwork
Yajima et al. 2023 [12]	A single institution study	13	30	Transient ischemic attack, infarction, and Headache	Not mentioned	37	Cerebral bypass	Regional anesthesia	Cesarean section: 9 women, Vaginal delivery: 4 women	Patients with Moyamoya disease experience a higher frequency of hypertensive disorders compared to normal pregnancies
Jeong et al. 2022 [13]	Research article	412	30.5	Cerebral infarction: 17, intracerebral hemorrhage: 37, subarachnoid hemorrhage: 6	Not mentioned	Not mentioned	Not mentioned	Not mentioned	Cesarean section: 259 women	The incidence of cerebrovascular disease increases after delivery in women with Moyamoya disease
Inayama et al. 2019 [14]	Retrospective review of cases	20	35	Ischemia: 17, hemorrhage: 2, seizure: 1	Single-photon computed tomography	38	Cerebral bypass	Not mentioned	Cesarean section: 10 women, vaginal delivery: 10 women	Intracranial hemorrhage, particularly at 24 weeks, is common during the antepartum period, while cerebral infarction is more common postpartum
Jung et al. 2015 [15]	Original article	28	31.9	Transient ischemic attack: 12, hemorrhage: 7, ischemia: 3	Not mentioned	38	Cephalo-duro-arterio-synangiosis (EDAS) and external ventricular drainage (EVD)	Regional anesthesia: 19, general anesthesia: 6	Cesarean section: 25, vaginal delivery: 2	Pregnant women with MMD should manage blood pressure, prevent hyperventilation, and choose the best delivery and anesthesia methods to avoid adverse postpartum outcomes
Takahashi et al. 2012 [16]	Original article	554	43.1	Transient ischemic attack: 63, hemorrhage: 33	Not mentioned	38	Bypass surgery	Not mentioned	Cesarean section: 117, vaginal delivery: 220	Moyamoya disease diagnosis reduces perinatal neurological events, but serious complications, particularly intracranial hemorrhage, can occur if the disease is not detected during pregnancy

This case study emphasizes the rare manifestation of MMD in pregnant individuals. Further research is crucial to enhance our comprehension of the underlying pathology and triggers of this complex ailment. Moreover, randomized controlled trials will be necessary in the coming years to discover new treatment approaches that might minimize complications both during and after pregnancy, ultimately decreasing the number of women affected by this condition and related anxiety. This is believed to be the first MMD appearance during pregnancy in Greece.

## Conclusions

This case demonstrates an uncommon occurrence of Moyamoya disease (MMD) during pregnancy in Europe, particularly in Greece. The positive outcomes for both the mother and newborn were the result of prompt diagnosis, careful multidisciplinary coordination, and the choice to conduct a planned cesarean section. Existing literature indicates that women with MMD are at a heightened risk for ischemic and hemorrhagic incidents during pregnancy and the postpartum period, highlighting the necessity for personalized care and rigorous hemodynamic management. While cesarean delivery is typically preferred to reduce neurological hazards, there is a notable lack of evidence-based protocols for optimal management during pregnancy. This situation emphasizes the urgent requirement for prospective, multicenter research to establish standardized guidelines that can enhance the safety and results for both the mother and her child.
